# New indices for home nursing care resource disparities in rural and urban areas, based on geocoding and geographic distance barriers: a cross-sectional study

**DOI:** 10.1186/s12942-015-0021-9

**Published:** 2015-10-08

**Authors:** Shyang-Woei Lin, Chia-Feng Yen, Tzu-Ying Chiu, Wen-Chou Chi, Tsan-Hon Liou

**Affiliations:** Department of Natural Resources and Environmental Studies, National Dong Hwa University, Hualien, Taiwan; Department of Public Health, Buddhist Tzu-Chi University, Hualien, Taiwan; Institute of Medical Sciences, Tzu Chi University, Hualien, Taiwan; School of Occupational Therapy, Chung Shan Medical University, Taichung, Taiwan; Department of Physical Medicine and Rehabilitation, Shuang Ho Hospital, Taipei Medical University, Taipei, Taiwan; Graduate Institute of Injury Prevention and Control, Taipei Medical University, Taipei, Taiwan

**Keywords:** Geographic information system, Long-term care, Home nursing care, Disparities, WHODAS 2.0

## Abstract

**Background:**

Aging in place is the crucial object of long-term care policy worldwide. Approximately 15.6–19.4 % of people aged 15 or above live with a disability, and 15.3 % of them have moderate or severe disabilities. The allocation of home nursing care services is therefore an important issue. Service providers in Taiwan vary substantially across regions, and between rural and urban areas. There are no appropriate indices for describing the capacity of providers that it is due to the distances from care recipients. This study therefore aimed to describe and compare distance barriers for home nursing care providers using indices of the “profit willing distance” and the “tolerance limited distance”.

**Methods:**

This cross-sectional study was conducted during 2012 and 2013 using geocoding and a geographic information system to identify the distance from the providers’ locations to participants’ homes in urban (Taipei City) and rural (Hualien County) areas in Taiwan. Data were collected in-person by professionals in Taiwanese hospitals using the World Health Organization Disability Assessment Schedule 2.0. The indices were calculated using regression curves, and the first inflection points were determined as the points on the curves where the first and second derivatives equaled 0.

**Results:**

There were 5627 participants from urban areas and 956 from rural areas. In urban areas, the profit willing distance was 550–600 m, and we were unable to identify them in rural areas. This demonstrates that providers may need to supply services even when there is little profit. The tolerance limited distance were 1600–1650 m in urban areas and 1950–2000 m in rural areas. In rural areas, 33.3 % of those living inside the tolerance limited distance and there was no provider within this distance, but this figure fell to just 13.9 % in urban areas. There were strong disparities between urban and rural areas in home nursing care resource allocation.

**Conclusions:**

Our new “profit willing distance” and the “tolerance limited distance” are considered to be clearer and more equitable than other evaluation indices. They have practical application in considering resource distribution issues around the world, and in particular the rural–urban disparities for public resource.

## Background

According to the most recent World Report on Disability, from the World Health Organization (WHO), between 15.6 and 19.4 % of people aged 15 years or over live with a disability, and 15.3 % of those have moderate or severe disabilities [1]. The Census and Statistics Department of the Ministry of Health and Welfare in Taiwan indicates approximately 1.13 million people with disabilities were living in Taiwan at the end of March 2014, or 4.84 % of the total population, and 39 % were over 65 years old [2]. The disabled population in Taiwan is substantially increasing as the age of the population increases and morbidity is reduced with advances in medical science and public health. As the global population ages, problems related to long-term care (LTC) present a formidable challenge to public health and social welfare policy.

In Taiwan, there is a national LTC policy, which is the responsibility of the National Health Insurance Administration (NHI) and the National 10-Year Long-term Care Plan. This program began in 2007, and offers services to people over the age of 65 with limitations on daily living, those over 50 with identified disabilities, and aboriginal people over 55. The fees for services provided under the program are covered 70–100 % by government, with users co-paying 0–30 % of total fees [3, 4]. Home nursing care is supplied by NHI, as part of universal medical coverage, with fees covered by insurance. The majority of people under 50 with disabilities who required LTC services meet the qualifications for low-income households and receive limited healthcare services primarily through the NHI system. As this 10-year program ends, and due to the aging population and rising healthcare costs, the Taiwanese government plans to implement a social insurance LTC system for the whole population [4]. Aging in place is a crucial focus of LTC policy worldwide, and the allocation of home care services will be an important issue [5]. Many surveys have demonstrated that in-home care is a desirable alternative to institutional care settings. Most people prefer to receive care at home when recovering from an injury or receiving rehabilitation or treatment for a chronic condition or disability. In Taiwan, the three most frequently used public LTC services in 2011 were leases or subsidies for assistive devices, home care services, and home nursing care [6]. For people with more severe disabilities, particularly those involving the loss of functioning in self-care, mobility and cognition, the nursing care demand is expected to increase [5, 6].

Nursing care is the one of home care services that can be offered at home or in an institution, and nursing care services must be supplied by nurses or physicians in Taiwan. The qualifying requirements for nursing care at home under NHI are: (1) people with chronic disease, i.e., those with limited capacities for self-care and who are limited to a bed or chair for more than 50 % of their activities [60 points or less on the Barthel Index or three or more on the scale of the Eastern Cooperative Oncology Group (ECOG)] and (2) people receiving hospice palliative care. Nursing services provided as part of a home care package in Taiwan include interviews by nurses or physicians, basic treatment, care for stomas (respiratory, digestive and urinary tract), and medication. The 10-year LTC plan has more lenient requirements than NHI for provision of home nursing care. Program users are assessed by the local government’s LTC center, and may have moderate health status, such as moderate pressure ulcer or other disability of movement.

Home nursing care services are either free or require a co-payment of up to 30 %, depending on users’ household income. Providers of home nursing care services receive a “fee-for-service (fee by piece)” and are paid by the NHI or 10-year LTC plan. Users who live further away equate to higher costs for providers, but this is not recognized in the remuneration package, and is therefore a significant factor in providers’ willingness to supply services. LTC provisions in Taiwan vary substantially across regions, and there are also differences between rural and urban areas. Environmental barriers are important issues for the use of public resources, particularly national medical and social welfare resources. Whiteneck [7] noted that there is much to learn about the role of environmental factors in the lives of those with disabled process, and researchers need to develop theories that underlie concepts to help to identify the conditions in which barriers actually prevent participation. For those with health and social care needs, social justice is influenced by environmental factors.

Benefits from the development of information technology and the rapid accumulation of digital data can be observed everywhere. Public health also provides a large and growing record of spatial information. This large volume of digital data, however, is spread across different government agencies and is difficult to obtain. Complex databases relevant to broader goals are difficult to understand and exponentially increase the inconvenience of preservation but might also hide much important knowledge. Geographical information systems (GIS) can aid in understanding and mining this data and in knowledge discovery by providing a platform for evaluating, managing, and integrating data in a geographic framework.

Geocodings are queries about addresses and places that convert information to coordinates used to plot maps and perform GIS analyses. Syu [8] used GIS to explore the medical resource distribution in the east area of Taiwan, including the remote areas of Hualien and Taitung County, where the population density is about 71.78 and 63.44 people/km^2^ (332,242 people/4628.6 km^2^, 223,025 people/3515.3 km^2^). He found that the profit or benefit obtained from providing care is an important factor for providers. We sought to understand whether there are profitable distances between providers and users and the extent to which policy should be involved if the distance between the two exceeds the tolerance limit of the providers. A prerequisite for meeting these goals is to examine regression curves obtained from the geocoding of the distance from users to the nearest provider, and employ scientific measures to discuss the influence of the barriers between providers and users in urban and rural areas.

The purpose of this study was therefore to describe and compare the distance barriers of providers using concrete indices describing “profit willing distance” and the “tolerance limited distance”. These indices were calculated based on the distances between users and providers in Taipei City and Hualien County. These findings should advance discussion about the disparities in the home nursing care supply. These new indices may also have practical applications and value in public resource distribution issues around the world, particularly in considering rural–urban disparities.

## Methods

The cross-sectional study was applied and approved by the Research Ethics Committee of Hualien Tzu Chi Hospital, Buddhist Tzu Chi Medical Foundation (IRB102-178). We assessed the level of function among older people with disabilities using the Chinese version of the World Health Organization Disability Assessment Schedule 2.0, 36-item version (WHODAS 2.0-36) [9]. Data were collected from 239 Taiwanese hospitals authorized to evaluate disabilities from July 2012 to October 2013 by occupational therapists, physical therapists, speech therapists, social workers, psychologists, and nurses. Address data for home nursing care providers were collected from the Ministry of Health and Welfare in 2014, as these providers must be formally registered. There were 33 home nursing care providers in Taipei City and 19 in Hualien County whose services included nursing care for older people with loss of functioning in self-care or needs or requiring treatment and care by nurses and physicians [2].

### Participants

The population included all adults with disabilities over the age of 50 years old who were officially registered in the former Disability Eligibility Determination System of Taiwan, and who lived in either Taipei City or Hualien County. Anyone living in an institution was excluded from the sample. After evaluation using the traditional Chinese version of WHODAS 2.0 36-item version, the study included 6583 potential participants, 5627 from Taipei City and 956 in Hualien County. The inclusion criterion was a functioning score in the “Self-care” domain of the survey instrument that was greater than the population mean score (≥ 39.93), in line with the requirements of NHI and 10-year LTC plan; higher scores indicate more severe dysfunction (score ranges for all domains: 0–100). The most common diagnoses among the participants were stroke and cerebral vascular disease (about 30 %, ICD 430–440.9) and dementia (about 24 %, ICD 290.0–290.9 and 298.9). Other diagnoses were nephrotic syndrome (ICD 581–590) and Parkinson’s disease, Paralysis agitans (ICD 332.0, 332.1).

All participants or their proxies were volunteers and provided written informed consent before being assessed. Each participant was assessed in person by a qualified tester. When a participant was identified as having a mental impairment or a communication disability and was unable to answer the questions by themselves, a proxy interview was performed with their primary caregivers.

Urban and rural areas were defined using the definition of urbanization published by Taiwan’s National Health Research Institutes [10], based on the following variables: population density (people/km^2^), ratio of people with college or greater educational levels in the population, ratio of the population over the age of 65 years old, ratio of the population in agriculture work, and number of physicians per 100,000 people. Using these definitions, Taipei City, the capital of Taiwan, was considered to be urban and Hualien County rural.

### Chinese version of WHODAS 2.0, 36-item version

The 36-item version of WHODAS 2.0 was developed using the WHO’s 2010 International Classification of Functioning, Disability and Health (ICF). This aims to measure the activities and participation in daily living within the previous 30 days in each of the following six domains: (1) cognition, with six items including concentrating, remembering, problem-solving, learning and communicating; (2) getting around, five items including standing, moving around inside the home, getting out of the home and walking a long distance; (3) self-care, four items covering hygiene, dressing, eating and staying alone; (4) getting along with people, with five items including interactions with other people and difficulties that might be encountered due to a health condition; (5) life activities (household and school/work), with eight items related to difficulties with day-to-day activities (i.e., activities that people perform on most days, including those associated with domestic responsibilities, leisure, work and school); and (6) participation, eight items covering social dimensions, such as community activities, barriers and hindrances to participation, and problems with other issues such as maintaining personal dignity. The possible responses to each item are mild, moderate, severe, extreme and no difficulty [11, 12]. The total score range was 0–100, and higher scores indicate greater levels of disability. The items related to employment and studying were excluded, leaving 32 items. The Chinese WHODAS 2.0, 36-item version was developed and published in 2013–2014 in Taiwan and has shown good validity and reliability [9].

### The index definitions and data analysis

The data were analyzed using the Statistical Package for the Social Sciences (v20.0, SPSS, Chicago, IL, USA) and the Geocoding and Geographic Information System (ArcGIS 9.3). Differences in demographic variables between Taipei City and Hualien County were evaluated using the Chi square test and Fisher’s exact test. We also used regression to define the indices for home nursing care supply (see below). The differences between the groups were considered significant when the p-values were below 0.05.

Geocoding and a geographic information system were used to convert address records into XY coordinates. Every point was the result of a specific spatial process that involves the minimum ZIP administrative center for ethical reasons. The data were plotted on a digital map and used in the spatial analysis to understand the effects of distances from providers to particulars.

**Fig. 1:**
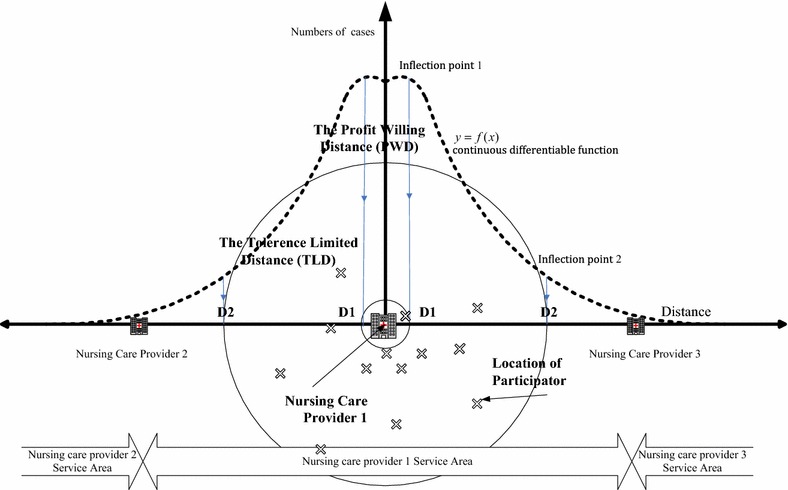
The definitions of “profit willing distance (PWD)” and the “tolerance limited distance (TLD)”

The definition of profit- willing distance (PWD) is the capacity and willingness of providers to supply services at different distances. We divided the participants’ distance from home nursing care providers into 50-m groups and used regression analysis to identify the first significant inflection point (inflection point 1 in Fig. 1). The “PWD (D1 in Fig. 1)” was then defined as the distance with the maximum number of participants. The “tolerance limited distance (TLD)” (D2 in Fig. 1) was defined as the distance between providers and participant which is over the providers’ burden to supply. That indicating the participants’ distance is too long to serve by the providers. We then computed the distances from the significant inflection point (inflection point 2 in Fig. 1) to locations.

The inflection points were determined by a continuous differentiable function, and could potentially occur more than once. Inflection points 1 and 2 were the first occurrence and the nearest distance from the origin to that point. For inflection point 1, we assumed that $y = f(x)$ is a continuous differentiable function. If there is a $\delta > 0$ that makes the graphic function $({\text{D}}1 - \delta ,{\text{D}}1)$ slope > 0 and $({\text{D}}1,D1 + \delta )$ slope < 0, that was defined as the point of the first derivative $f^{'} (D1) = 0$. We termed $(D1,f(D1))$ of the f continuous function “inflection point 1”. For inflection point 2, if the pattern of convexity in $({\text{D}}2,f({\text{D}}2))$ changed to make the graphic function in $({\text{D2}} - \delta ,{\text{D}}2)$ concave in a downwards direction and that of $({\text{D}}2,{\text{D}}2 + \delta )$ concave in an upwards direction, the point of the second derivative f^′′^ before D2 was positive, and f^′′^ after D2 was negative. That is the point of second derivative $f^{\prime\prime}({\text{D}}2) = 0$, which we called $({\text{D}}2,f({\text{D}}2))$ in the f continuous function “inflection point 2”.

## Results

### Sample characteristics

The study included 5627 participants in the urban area and 956 in the rural. The mean ages were 75.6 and 72.9 years, and the percentage of females in the urban area was greater (53.5 vs. 48 %). There were significant differences in age and gender between the two locations (p < 0.05).

According the classifications of “body function and structure” of the ICF, the principal impairment types were “neuromusculoskeletal- and movement-related functions” for our participants in both cities (46 and 57.2 %), and there were no differences in any categorythe percentage of impaired participants in the rural area was significantly higher (p < 0.05). The moderate level of severity was the most disparate between the groups. The top two domains of limitations were “life activity” (mean score = 79.3 ± 33.1) and “getting along” (mean score = 70.0 ± 30.8) in the urban area and “life activity” (mean score = 79.6 ± 30.7) and “moving” (mean score = 66.3 ± 27.8) in the rural area. There were significant differences in “cognition”, “moving”, “self-care”, “getting along”, and “life activity for work” (p < 0.05, Table 1).

**Table 1:** The demographic and health characteristics of the samples

Variables	Taipei (N = 5627)	Hualien (N = 956)	Values of chi-square/t-test
	n (%)	n(%)	
Age			
Mean ± SD	75.61 ± 12.0	72.88 ± 11.7	6.53***
Gender			
Male	2618 (46.5)	497(52)	9.78**
Female	3009 (53.5)	459 (48)	
Impairment of body function and structure			
Mental functions and structure of the nervous system	2470 (43.9)	325 (34)	32.78***
Sensory functions and pain	653 (11.6)	95 (9.9)	2.26
Voice and speech functions	89 (1.6)	18 (1.9)	0.46
Functions of the cardiovascular, hematological, immunological and respiratory systems	302 (5.4)	53 (5.5)	0.05
Functions of the digestive, metabolic and endocrine system	234 (4.2)	30 (3.1)	2.21
Genitourinary and reproductive functions	384 (6.8)	82 (8.6)	3.82
Neuromusculoskeletal and movement related Functions	2587 (46.0)	547 (57.2)	41.41***
Functions of the skin and related structures	6 (0.1)	1 (0.1)	0
Severity of disability^a^			
Mild	1206 (21.4)	193 (20.2)	8.133*
Moderate	1888 (33.6)	288 (30.1)	
Serious	1031 (18.3)	203 (21.2)	
Profound	1502 (26.7)	272 (28.5)	
Functioning of activity and participation			
Cognition	59.44 ± 32.18	52.70 ± 35.35	5.52***
Mobility	61.68 ± 28.67	66.25 ± 27.78	−4.57***
Self-care	43.15 ± 32.18	49.26 ± 34.21	−5.15***
Getting alone	66.99 ± 30.77	55.66 ± 38.32	8.68***
Life activity	79.29 ± 33.13	79.62 ± 30.74	−0.31
Life activity (Work)	35.69 ± 47.46	58.14 ± 49.22	−13.11***
Participation	53.86 ± 23.38	54.23 ± 24.04	−0.45
Summary scores	61.52 ± 20.58	61.52 ± 21.35	−0.002

* p < 0.05, ** p < 0.01, *** p < 0.001

^a^The evaluation was according to body function and body structure of the ICF from the Functioning Disability Evaluation Scale-Adult Version (FUNDES-adult) of Taiwan [15]

### Indices for home nursing care

**Fig. 2:**
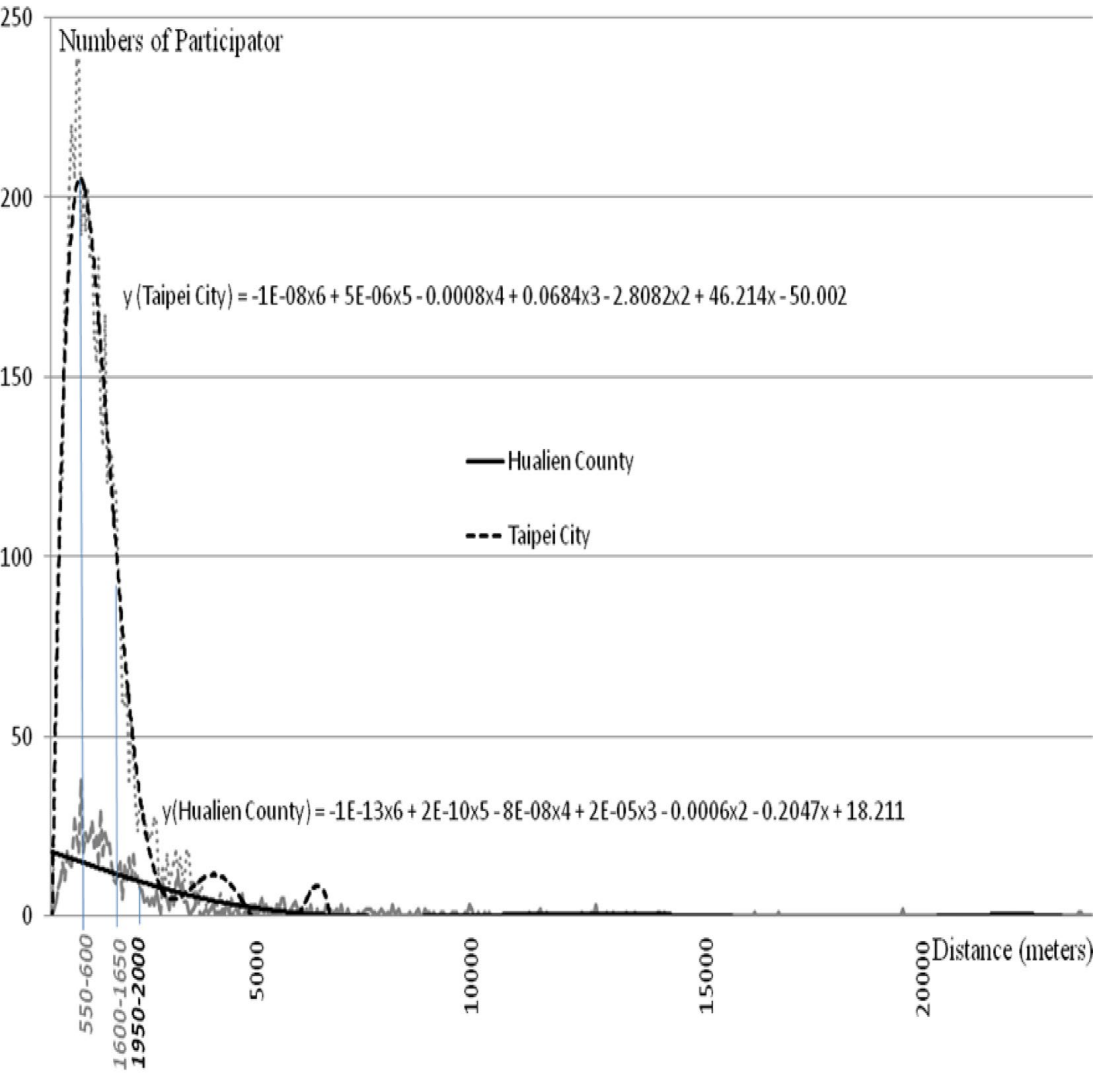
The trend line distance between the participants and providers

The regression analysis (Fig. 2) indicated that inflection point 1, the PWD, was located in the 550–600-m group for Taipei City. Inflection point 2 for Taipei City, or the TLD, occurred in the group living 1600–1650 m from the home care nursing provider. We were unable to identify inflection point 1 from the regression for Hualien County, which indicated that the PWD was not detected by regression. Inflection point 2, the TLD, occurred in the 1950–2000-m group.

### Disparities in nursing care at home

**Fig. 3:**
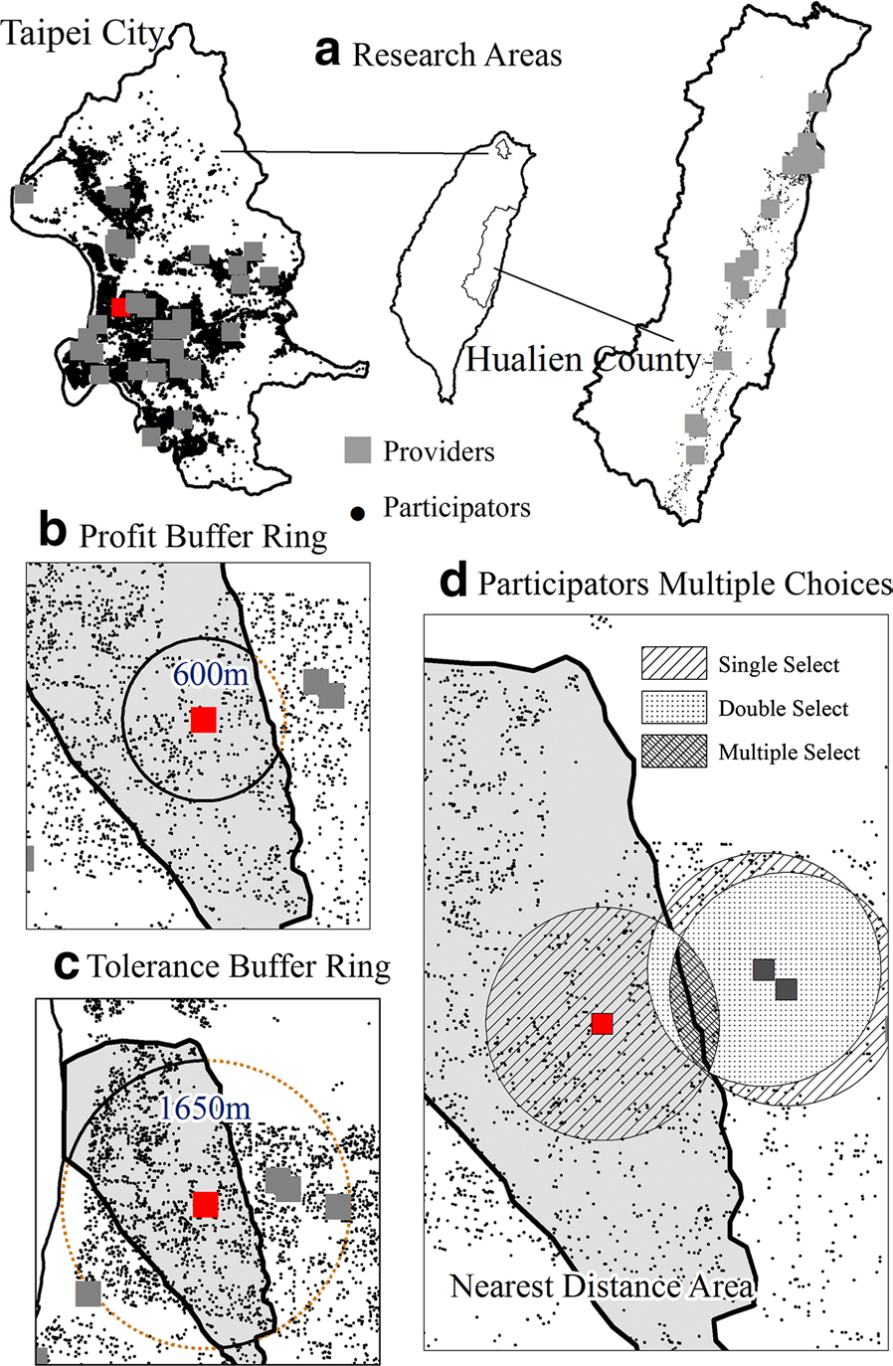
The diagrams of providers buffer rings and participators multiple choices. **a** Locations of the participants in Taipei City and Hualien County. **b** Profit buffer ring of Taipei City. **c** Tolerance buffer ring of Taipei City. **d** Overlapping area of Taipei City and the participants multiple choices

There were 33 home care nursing providers in Taipei City and 19 in Hualien County (see Fig. 3a). We counted the numbers of participants and providers at the PWD and TLD for each location, as symbolic representations of the numbers of providers that could be chosen by the participants (Fig. 3). For example, using the PWD (600 m) and TLD (1650 m) as the buffer rings for every provider in Taipei (Fig. 3b, c) and illustrating every buffer ring for all providers, the overlapping areas in Fig. 3d indicate the choice available to participants. Finally, we calculated the numbers of participants with one, two and more possible providers, and the results are shown in Table 2.

**Table 2:** The numbers of nursing care providers that could be selected within the profit willing distance (PWD) and tolerance limited distance (TLD)

Participants share care providers, (n)	Hualien (n, %)		Taipei (n, %)	
Within PWD				
Without any care center	–	–	3791	67.37 %
≥1	–	–	1836	32.63 %
1	–	–	1501	81.75 %
2			270	14.71 %
3	–	–	65	3.54 %
Total number of Participants	956	100.00 %	5627	100.00 %
Within TLD				
Without any care center	318	33.26 %	780	13.86 %
≥1	638	66.74 %	4847	86.14 %
1	334	52.35 %	1218	25.13 %
2	198	31.03 %	1113	22.96 %
3	61	9.56 %	1091	22.51 %
4	44	6.90 %	728	15.02 %
5	1	0.16 %	376	7.76 %
6	0	0.00 %	234	4.83 %
≥7	0	0.00 %	87	1.79 %
Total number of participants	956	100.00 %	5627	100.00 %

–, not available

Within the PWD (600 m) in Taipei City, 67.4 % of the older people with disabilities did not have access to multiple home nursing care providers. Of the remaining 32.6 %, approximately 3.5 % had access to three possible providers.

There was no PWD for Hualien County. Although the TLD was larger in Hualien County than in Taipei City (1950–2000 vs 1600–1650 m), 33.3 % of participants living inside the tolerance distance and there was no provider within this distance. The gap between the two areas was significant, because 86.1 % of the participants in Taipei City could choose from more than one care provider (Table 2).

## Discussion

This study has provided new indices to examine disparities in resource allocation. The PWD and TLD can clearly show capacity limitations based on the distance burdens from providers to users. The results of our study about the numbers of providers that could be selected by the participants within the two distances help to explain the disparities in home care nursing services between rural and urban areas, and may do so better than the service density (i.e., the ratio of people with the needs to those with providers).

### Potential needs and supplies of home care nursing in Taipei City and Hualien County

Age and the severity of disability are the most important factors in determining the use of nursing care [6]. Based on the characteristics of our sample, we can therefore discuss the potential needs for home-care nursing in the two areas. We found that the sample from Taipei City was significantly older than that from Hualien County, but had a smaller percentage of participants with serious and profound disabilities. This suggests that the needs for home care nursing in the two areas should be similar. The percentage of participants with impairments in mental function and nervous system structure was higher in the urban area, but there were more physical impairments among rural residents. There were significant differences in the Moving and Self-care domains in the rural sample.

Many validation studies and clinical interventions have indicated that the WHODAS 2.0 is strongly correlated with both activities of daily living (ADL) and the Barthel Index [13–15]. The sample of rural residents showed more severe ADL and Barthel Index scores, and both of these measures are evaluation tools for home care nursing services in Taiwan [16]. This suggests that additional home care nursing services are needed for older people in Hualien County.

There were 33 home nursing care providers in Taipei City and 19 in Hualien County. The ratios of people with needs to providers (i.e., the service densities) were therefore 50 people/provider (956 samples/19 home-care nursing) in Hualien and 110 people/provider (33/5627) in Taipei. These figures suggest that the supply of home-care nursing was better in Hualien County. However, the total area of Hualien County is 4628.6 km^2^, much greater than that of Taipei City (271.8 km^2^), which shows that home care nursing services providers must travel greater distances in the rural area.

It is important to note, however, that the participants in our study were not necessarily users of home nursing care, only those who would qualify for its use, and this might grossly overestimate the demand for home nursing care. The same criteria are used to determine eligibility in both areas, however, and the severity of disability distribution was not significantly different, so this probably did not affect the results.

### Index of home nursing care services disparities

Chen and other researchers have used medical and healthcare expenses, the numbers of healthcare institutions, beds and healthcare professionals, and the health status of the population to compare healthcare disparities between urban and rural areas [17]. The distribution of healthcare, or accessibility or equity, for example, availability of healthcare professionals or the density of care services, have been used as indices to express resource disparities for children with disabilities, older people, patients with cancer and patients in urban and rural areas [18–20]. Specifically, Geographical information systems can be used as the primary tool to measure the distribution and accessibility of medical resources or the behaviors of health service utilities [19, 21–23], but no appropriate index for explaining the capacities of service providers in terms of distance has been developed. For home nursing care, the principal services must be supplied on site by physicians and nurses; willingness and capacity of providers are therefore important in promoting the health of patients. Our use of GIS to measure the distances between providers and participants and identify the first and second inflection points is similar to the method employed by Alexandra and Zachary (2007) and Chang et al. [19]. In the first, relevant resources were mapped to help to improve emergency management for people with disabilities in urban and rural areas in the United States. In the latter study, the authors demonstrated that the inflection points of the geographical accessibility of hospitals and travel patterns of patients were significant, and this information was applied to understand patients’ hospital-seeking behaviors in Taiwan. These authors did not, however, develop an index to express the possible barriers and burdens due to distance that are encountered by care service providers or compare resource allocation between urban and rural areas.

For providers or the government, the profitable distance is a key point for the provision of services to customers, especially for home care based on distance. In theory, the PWD would be a good index to determine the maximum distances at which providers are willing to supply services, although its theoretical applicability would need to be tested to examine whether the estimates are accurate. The inability to detect a PWD in some areas might indicate that those areas are not profitable, and there are therefore likely to be very few medical resources available.

The TLD indicates the limited distance at which providers will tolerate supplying services, but longer TLDs do not mean that a provider has greater capacity. For home care services under the NHI and social welfare system, the TLD means that providers must exert effort to supply services even if they do not make a profit in doing so. The expected result is that TLDs will be longer in more remote districts and areas with fewer resources.

The concepts of PWD and TLD are very important particularly in some countries, where home nursing care services or other medical services paid for by government do not include an element of funding for distance, the cost of which must therefore be borne by providers. This cost therefore influences providers’ decisions.

### Resource allocation between urban and rural areas

A larger proportion of older adults living in rural areas are likely to have less access to formal services such as hospitals, home care, physicians and other healthcare providers and may have to travel greater distances to access health services [24–30]. Chen et al. found that the numbers of healthcare institutions, beds and healthcare professionals were significantly different between urban and rural areas in China during 2000–2010 [17]. Rural residents also have more difficulty obtaining referrals for secondary or specialist medical care than urban residents, and these disparities pose significant barriers to care, particularly for older people and children [31, 32]. A small study of American Indian and Alaskan Native adults revealed that long travel distances are a significant barrier to the use of healthcare services among rural residents [33]. The distance between the user and provider, however, does not show the provider’s willingness to provide services over distances or the burden of doing so. We need a clearer index to discuss and compare the capacities of providers to supply the care afforded by the national health insurance and social welfare system, particularly those services that are provided in care recipients’ homes.

In the rural area in our study, we could not detect a PWD, perhaps because of the low density of users and their uneven geographical spread. It may be that the PWD cannot be detected in areas in which the resource distributions are not uniform, but it also seems likely that there is a surplus of resources in shorter PWD areas. In health or social care welfare resource allocation, the central government usually depends on the size of the population with needs within every administrative division such that smaller populations with disabilities tend to receive fewer resources. Distance barriers may therefore need to be considered in allocating resources. Regarding the distance burden of the official supply, the TLD is just sufficient to answer the question; longer TLDs indicate greater loads on the providers’ supplies.

Moreover, by using overlapping areas, we showed the theoretical ability of patients to choose from multiple providers, giving an indication of resource allocation. We found that a high percentage of the sample in the rural area were not able to choose providers within the buffer ring (Table 2). In brief, a longer TLD and a higher percentage of users without any providers to choose from were observed in Hualien County, and these findings indicate significant disparities in home nursing care from both the providers’ and users’ perspectives.

## Conclusion

Home care resource allocation exhibited strong disparities between urban and rural areas in Taiwan, and these disparities did not seem to be based on the proportion of the population in need or the ratio of providers to that population. We therefore developed two indices, which we called the profit-willing distance (PWD) and tolerance limited distance (TLD), which helped to express the burdens and capacities of providers. The PWD was not detected in Hualien, but the TLD was longer, which indicates that providers were more likely to supply home nursing care services in remote districts even when it was not profitable. The use of the numbers of possible providers within each distance to discuss the equity of services revealed that there were disparities between the urban and rural areas. Our findings could be importance references for evaluating LTC resource allocation and aiding decision-making for national LTC planning.
